# Preventable cancer cases and deaths attributable to deficit of physical activity in Korea from 2015 to 2030

**DOI:** 10.4178/epih.e2025010

**Published:** 2025-01-27

**Authors:** Soseul Sung, Sungji Moon, Jihye An, Jeehi Jung, Hyeon Sook Lee, Youjin Hong, Sangjun Lee, Woojin Lim, Kyungsik Kim, Inah Kim, Jung Eun Lee, Sun Ha Jee, Aesun Shin, Ji-Yeob Choi, Sun-Seog Kweon, Min-Ho Shin, Sangmin Park, Seungho Ryu, Sun Young Yang, Seung Ho Choi, Jeongseon Kim, Sang-Wook Yi, Yoon-Jung Choi, Jeong-Soo Im, Hong Gwan Seo, Sohee Park, Kwang-Pil Ko, Sue K. Park

**Affiliations:** 1 Department of Preventive Medicine, Seoul National University College of Medicine, Seoul, Korea; 2 Department of Biomedical Sciences, Seoul National University Graduate School, Seoul, Korea; 3 Cancer Research Institute, Seoul National University, Seoul, Korea; 4 Interdisciplinary Program in Cancer Biology, Seoul National University College of Medicine, Seoul, Korea; 5 Department of Epidemic Intelligence Service, Incheon Communicable Diseases Center, Incheon, Korea; 6 Department of Biomedicine & Health Science, The Catholic University of Korea, Seoul, Korea; 7 Incheon Public Health Policy Institute, Incheon, Korea; 8 Integrated Major in Innovative Medical Science, Seoul National University Graduate School, Seoul, Korea; 9 Department of Occupational and Environmental Medicine, Hanyang University College of Medicine, Seoul, Korea; 10 Department of Food and Nutrition, Seoul National University, Seoul, Korea; 11 Department of Epidemiology and Health Promotion, Institute for Health Promotion, Graduate School of Public Health, Yonsei University, Seoul, Korea; 12 BK21 Plus Biomedical Science Project, Seoul National University College of Medicine, Seoul, Korea; 13 Institute of Health Policy and Management, Seoul National University Medical Research Center, Seoul, Korea; 14 Department of Preventive Medicine, Chonnam National University Medical School, Hwasun, Korea; 15 Department of Family Medicine, Seoul National University Hospital, Seoul, Korea; 16 Department of Occupational and Environmental Medicine, Kangbuk Samsung Hospital, Sungkyunkwan University School of Medicine, Seoul, Korea; 17 Department of Internal Medicine, Healthcare Research Institute, Seoul National University Hospital Healthcare System Gangnam Center, Seoul, Korea; 18 Graduate School of Cancer Science and Policy, National Cancer Center, Goyang, Korea; 19 Department of Preventive Medicine and Public Health, Catholic Kwandong University College of Medicine, Gangneung, Korea; 20 Division of Cancer Registration and Surveillance, National Cancer Center, Goyang, Korea; 21 Clinical Preventive Medicine Center, Seoul National University Bundang Hospital, Seongnam, Korea

**Keywords:** Physical ativity, Exercise, Population-attributable fraction, Epidemiology, Korea

## Abstract

**OBJECTIVES:**

This study aimed to determine the population-attributable fractions (PAFs) of cancers using various calculation methods and to estimate the PAFs of cancer incidence and mortality resulting from deficit in physical activity (DPA) from 2015 to 2030, based on data on prevalence rates.

**METHODS:**

The PAF of cancer was estimated using a cohort study-based meta-analysis of relative risk (RR), national prevalence rates of DPA from 2000 to 2015, and national cancer statistics from 2015 to 2030, with a latency of 15 years.

**RESULTS:**

In 2015, DPA contributed to 909 cancer cases and 548 deaths, accounting for 0.42% and 0.68% of new cancer cases and deaths, respectively. By 2030, the PAF values are expected to increase to 1.31% of incidence and 1.80% of mortality, with a continual increase from 2015 to 2030. When the low metabolic equivalent of task (MET) criteria were selected, the PAF values decreased for both incidence and mortality. The PAF calculated with <900 MET-min/wk for the sex-specific MET criterion was higher than that calculated with <900 MET-min/wk for both incidence and mortality.

**CONCLUSIONS:**

The risk of cancer associated with DPA is expected to rise in both male and female. Future research and strategies should emphasize the promotion of physical activity for cancer prevention, considering its significant implications for public health.

## GRAPHICAL ABSTRACT

[Fig f5-epih-47-e2025010]

## Key Message

In 2015, deficit in physical activity (DPA) accounted for 0.42% of incident cancers (909 cases) and 0.68% of cancer deaths (548 deaths), with population-attributable fraction (PAF) values projected to rise to 1.31% for incidence and 1.80% for mortality by 2030. The cancer burden attributable to DPA is increasing in both sexes, underscoring the need to strengthen population- level physical-activity promotion for prevention.

## INTRODUCTION

Physical inactivity is closely associated with concerns such as obesity and being overweight, as well as excessive intake of fats and sugary foods, both of which contribute to cancer onset [1,2]. A deficit in physical activity (DPA) leads to reduced energy expenditure following food consumption, which can result in obesity or being overweight. The development of technological advancements in performing household chores, along with the emergence of innovative tools and devices such as cars, televisions, computers, and gaming consoles, has shifted lifestyles toward more sedentary behaviors, thereby exacerbating DPA

The metabolic equivalent of task (MET) is a crucial metric for evaluating physical activity (PA). One MET corresponds to the energy expenditure rate during restful sitting. Sedentary activities, such as office work, driving, and watching TV, generally require about 1.5 METs. Activities are classified by intensity: light PAs, which include walking, seated fishing, vacuuming, driving, and bowling (both practice and competition), require less than 3 METs; moderate PAs, such as brisk walking, doubles tennis, fishing while standing, and household chores like wiping and cleaning, require 3-6 METs; and vigorous PAs, including running, singles tennis, and moving furniture, require 6 METs or more [1-3]. The measurement of METs can be conducted by quantifying weekly PA in terms of frequency and intensity, or by incorporating the duration of the exercise into the MET calculation, which establishes a minimum time criterion. Additionally, tools like heart rate monitors and accelerometers provide accurate measurement options [1-3].

According to expert summaries from the International Agency for Research on Cancer (IARC) of the World Health Organization (WHO), the World Cancer Research Fund/American Institute for Cancer Research (WCRF/AICR), and the 2018 Physical Activity Guidelines for Americans (PAGA), there is compelling evidence that increased PA can reduce the risk of several cancers. Therefore, adopting a physically active lifestyle is recommended for cancer prevention. This research aimed to determine the influence of PA in the year 2000 on cancer incidence and mortality rates in 2015, and to estimate the trend in the DPA-related population-attributable fraction (PAF) among Koreans by 2030.

## MATERIALS AND METHODS

The International Physical Activity Questionnaire (IPAQ) guidelines recommend a minimum PA level of at least 600 MET-min/wk for adults [4]. The WHO guidelines for PA for adults aged 18-64 years suggest at least 150 minutes of moderate-intensity aerobic PA per week, or a minimum of 75 minutes of vigorous-intensity aerobic PA. For additional health benefits, adults may engage in more than 300 minutes of moderate-intensity or more than 150 minutes of vigorous-intensity aerobic PA per week, equivalent to over 900 MET-minutes [5]. In the United Kingdom, the impact of DPA on cancer was evaluated under the assumption that PA levels below 15 MET-hr/wk (less than 900 MET-min/wk) are associated with an increased risk of cancer [6]. Similarly, in France, DPA levels were defined based on cancer risk scenarios for activities below 21 MET-hr/wk (less than 1,260 MET-min/wk) and below 10.5 MET-hr/wk (less than 630 MET-min/wk) [7]. The thresholds of 900 MET-min/wk and 1,260 MET-min/wk correspond to engaging in moderate-to-vigorous PA at 6 METs for 30 minutes daily over 5 days and 7 days, respectively.

In this study, we initially assessed the contribution of DPA at the population level by setting the DPA threshold at <900 METs min/wk, consistent with previous standards used in the United Kingdom. Additional criteria for DPA included the minimum PA standards from the IPAQ (600 METs min/wk) and the criteria employed in France (1,260 and 630 MET-min/wk). Given that the average MET varies by sex, the threshold for DPA was established at 900 METs min/wk. This included a calculation of sex-specific METs and incorporated criteria for vigorous activity in premenopausal female.

The exposure rate of DPA was derived from the Korea National Health and Nutrition Examination Survey (KNHANES) [8]. Typically, KNHANES utilizes the IPAQ questionnaires to evaluate the types and weekly durations of PA, as seen in the data from 2005 and 2007-2020. However, the questionnaires from 1998 and 2001 differed, and no data were collected between 2002 and 2004. To compensate for these discrepancies, our study estimated the DPA rates for 2000 and 2005 using the 2005 and 2007 KNHANES data, which were standardized to the 2000 population. This adjustment involved using the 2005 data as a proxy for 2000 and the 2007 data for 2005. The DPA rates for 2010 and 2015 were directly based on the KNHANES data from those respective years.

In 2001, an expert group from the IARC of the WHO provided substantial evidence linking PA with the prevention of colon and breast cancer. However, they found only limited evidence for its impact on prostate and endometrial cancers [3,9,10]. The WCRF/AICR has determined that colorectal cancer (International Classification of Diseases, 10th revision [ICD-10] codes C18-C20) meets their criteria for a convincing level of causality, which includes considerations of causality, biological plausibility, and a dose-response relationship. Additionally, the organization has classified endometrial and postmenopausal breast cancers as having probable causality. They also indicated that vigorous PA could reduce the risk of breast cancer in female regardless of menopausal status [11]. Other cancers, such as esophageal, premenopausal breast, liver, and lung cancers, have been assigned a limited grade of causality [11].

In this study, we selected colorectal (ICD-10 codes C18-C20), postmenopausal breast (ICD-10 code C50), and corpus uteri cancers (ICD-10 code C54) to estimate the contribution of DPA. These cancers were chosen based on the WCRF/AICR’s evaluation, which provided either convincing or probable grades of strong causal evidence that PA reduces risk [9-11] (Supplementary Material 1). Although the WCRF/AICR identified strong causal evidence of a probable grade for vigorous PA across all menopausal statuses, our study included all types of PA for postmenopausal breast cancer and considered vigorous PA for premenopausal female in the sensitivity analysis. We calculated the cancer risk per 1 MET hour increase per week by sex and then converted this to relative risk (RR) per 1 MET-hr/wk reduction. Subsequently, we applied the natural logarithm to compute the beta. The RR of cancer by weekly MET-minute increments was determined through a meta-analysis (random-effect model) using raw data from the Korean Cohort Consortium [12-23].

Based on the 15-year latency period [24] and the consistent RR between exposure and outcome, the PAF of cancer due to DPA was calculated using the exposure rates in Korea for the years 2000, 2005, 2010, and 2015, along with the cancer incidence and mortality rates for the years 2015, 2020, 2025, and 2030, respectively. The number of new cancer cases and deaths among adults aged 20 and older was obtained from cancer registration and death statistics [16]. Projections for the population and the expected numbers of cancer cases and deaths for the years 2025 and 2030 have been previously described [25].

The PAF was differentiated by sex and calculated using equation (1) for the DPA dose on a continuous scale, modified from Levin’s formula. The 95% confidence intervals (CIs) for the PAF were determined using the Monte Carlo method [26-29].


(1) $$PAF=\frac{P_{e}\left(e^{\beta \cdot \text{dose}}-1\right)}{P_{e}\left(e^{\beta \cdot \text{dose}}-1\right)+1}$$

### Ethics statement

This study was approved by the Institutional Review Board of Seoul National University Hospital (IRB No. C-1911-188-1084).

## RESULTS

According to the minimum PA guideline of 900 METs min/wk, 23.6% of male and 28.5% of female were classified as physically inactive in Korea in 2000. Assuming a standard of moderate PA at 1,260 METs min/wk, the rate of inactivity increases when considering those taking less than 1,260 METs min/wk (male: 34.9%; female: 39.5%). The prevalence rate of DPA was projected to rise from 2000 to 2015 for both male and female (Supplementary Material 2).

In Korean cohort studies, the risk of cancer incidence and mortality was found to decrease with every 1 MET increase in PA. However, all 95% CIs included a value of 1.00, with the exception of female colorectal cancer deaths. Vigorous PA was linked to a 5% reduction in breast cancer incidence, whereas PA in postmenopausal female was associated with a 1% reduction in breast cancer incidence and a 2% reduction in breast cancer deaths (Supplementary Materials 3 and 4).

In 2015, the PAF for cancers due to DPA was 2.43%, 1.62%, and 3.22% for colorectal, breast, and corpus uteri cancer, respectively. For male, the PAF for colorectal cancer was 0.98% for incidence and 2.31% for mortality. For female, the PAF values for cancer incidence were 3.22% for colorectal, 1.62% for breast, and 3.22% for corpus uteri cancer. Regarding cancer-related deaths in female, the PAF values were 9.60% for colorectal, 3.86% for breast, and 8.99% for corpus uteri cancer (Table 1, Supplementary Material 5).

In 2015, out of the 909 cancer cases attributed to DPA, 658 were colorectal cancer cases, with 303 occurring in male and 355 in female. DPA was responsible for 1.88% of male colorectal cancer cases and 3.22% of female colorectal cancer cases. Additionally, of the 548 cancer deaths attributed to DPA that year, 454 were due to colorectal cancer, with 109 male and 345 female affected. Among these, 2.31% of male deaths and 9.60% of female deaths from colorectal cancer were attributed to DPA (Table 2, Supplementary Material 6).

In 2015 and 2020, the most common individual cancer cases attributable to DPA were colorectal (72.4 and 76.4%), breast (19.0 and 17.7%), and corpus uteri cancer (8.6 and 5.8%). For female, the percentages were colorectal (58.6 and 69.6%), breast (28.5 and 22.8%), and corpus uteri cancer (12.9 and 7.6%). Among cancer-related deaths attributable to DPA, colorectal cancer was the most prevalent (82.7 and 81.3%), followed by breast (12.0 and 12.7%), and corpus uteri cancer (5.3 and 6.0%). Specifically for female, the attributable deaths were colorectal (78.4 and 76.4%), breast (15.0 and 16.2%), and corpus uteri (6.6 and 7.4%) (Supplementary Materials 7-10).

**Figure 1.**
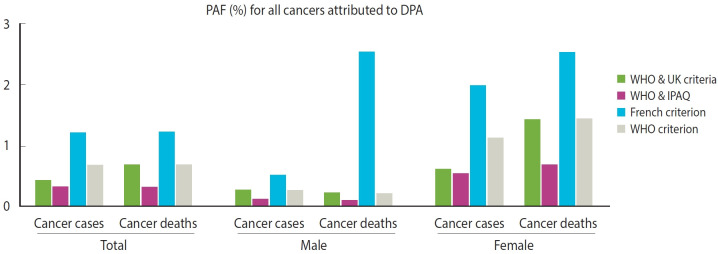
Comparison of cancer PAF attributed to DPA when using different relative risks. PAF, population attributable fraction; DPA, deficit in physical activity; WHO, World Health Organization; IPAQ, International Physical Activity Questionnaire.

Using point estimates related to the contribution rate of a 1 MET increase in PA and referencing the minimum PA standard of 900 METs min/wk, the contributions of DPA to cancer incidence and mortality were estimated at 0.42% and 0.68%, respectively. When the DPA criteria were set low, the PAF value also decreased due to a reduction in prevalence. Vigorous activity in premenopausal female was excluded from the analysis because its inclusion led to an overestimation of both the RR and the contribution of DPA (Tables 1 and 2 and Figure 1, Supplementary Material 11).

Using 1,260 METs min/wk criterion, the contributions to total population incidence and mortality were 1.20% and 1.21%, respectively. For male, these figures were 0.50% for incidence and 0.41% for mortality, while for female, they were 1.97% for incidence and 2.51% for mortality. With the criteria of 630 METs min/wk and 600 METs min/wk, these values decreased (at 630 METs min/wk, both incidence and mortality were 0.35%; at 600 METs min/wk, both were 0.32%). Using 900 METs min/wk with sex-specific MET calculations, the contributions for male were 0.26% (incidence) and 0.21% (mortality), and for female, they were 1.11% (incidence) and 1.43% (mortality) (Table 2, Supplementary Material 5).

**Figure 2.**
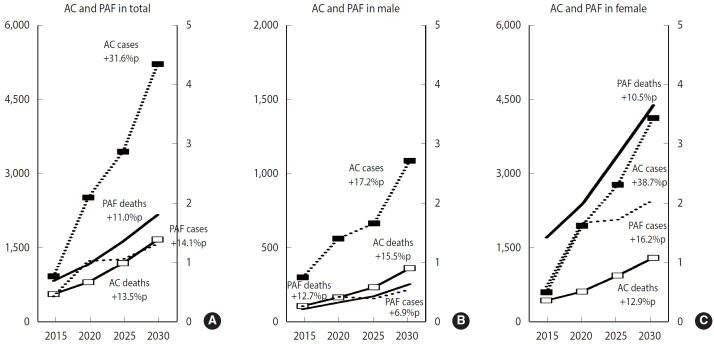
Changing trends of population attributable fraction (PAF) and attributable cancer cases and deaths (ACs) in cancer attributed to deficit in physical activity in Korea, 2015 to 2030 (A) total, (B) male, and (C) female. %p, percentage point.

**Figure 3.**
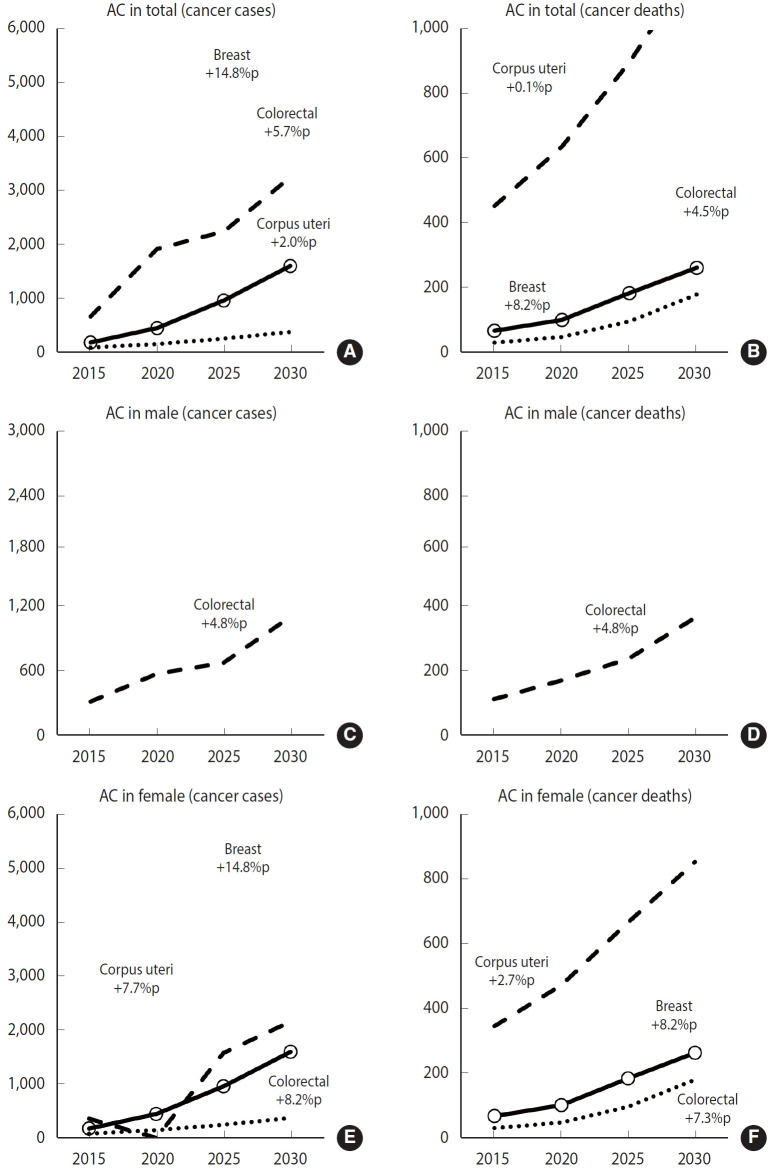
Changing trends of attributable cancer cases and deaths (ACs) in specific cancer attributed to deficit in physical activity in Korea, 2015 to 2030. Attributable cancer cases in (A) total, (C) male, (E) female. Attributable cancer deaths in (B) total, (D) male, and (F) female. %p, percentage point.

The PAF of cancer attributable to DPA is expected to increase consistently across the total population and among male from 2015 to 2030. Specifically, the PAF for the total population is projected to rise from 0.42% in 2015 to 1.31% in 2030, and for male, from 0.27% in 2015 to 0.55% in 2030. Similarly, the PAF for male deaths is forecasted to continuously increase from 0.22% in 2015 to 0.64% in 2030. Additionally, the PAF values for both incidence and mortality due to DPA in female are expected to rise steadily from 2015 to 2030, with the incidence PAF increasing from 0.60% in 2015 to 2.06% in 2030, and the mortality PAF from 1.42% in 2015 to 3.65% in 2030 (Figures 2 and 3, Supplementary Material 6).

## DISCUSSION

Using 900 METs min/wk as the threshold for adequate weekly PA, 0.90% of the total cancer incidence and 0.90% of the total cancer mortality among Koreans can be attributed to insufficient DPA. When this threshold is increased to 1,260 METs min/wk, the PAFs change to 1.75% for cancer incidence and 1.71% for cancer mortality, respectively.

A comparison with a 2009 study on the cancer contribution rate in Korea revealed significant changes over time [30,31]. By 2015, the contribution of DPA to colorectal cancer incidence in male had increased markedly (0.78% in 2009 vs. 1.88% in 2015, using the 900 METs min/wk criterion). Overall, the contribution of DPA to cancer in male rose from 0.10% in 2009 to 0.27% in 2015, based on the same criteria. For female, there was a notable increase in the contribution rate for most cancers, with the exception of breast cancer incidence and mortality. Specifically, the contribution rate to colorectal cancer incidence climbed from 0.87% in 2009 to over 3.22% in 2015, and for colorectal cancer mortality, it surged from 0.87% in 2009 to 9.60% in 2015, using the 900 METs min/wk criterion. Conversely, breast cancer saw a decrease in its contribution rate in 2015 compared to 2009 (incidence: 8.81% in 2009 vs. 1.62% in 2015; mortality: 8.81% in 2009 vs. 3.86% in 2015) [30,31] (Supplementary Material 12). An analysis of the trends in PA exposure from 2000 to 2015, along with projected cancer incidence and mortality rates from 2015 to 2030, indicated an upward trend from 2015 to 2030 for both incidence and mortality.

**Figure 4.**
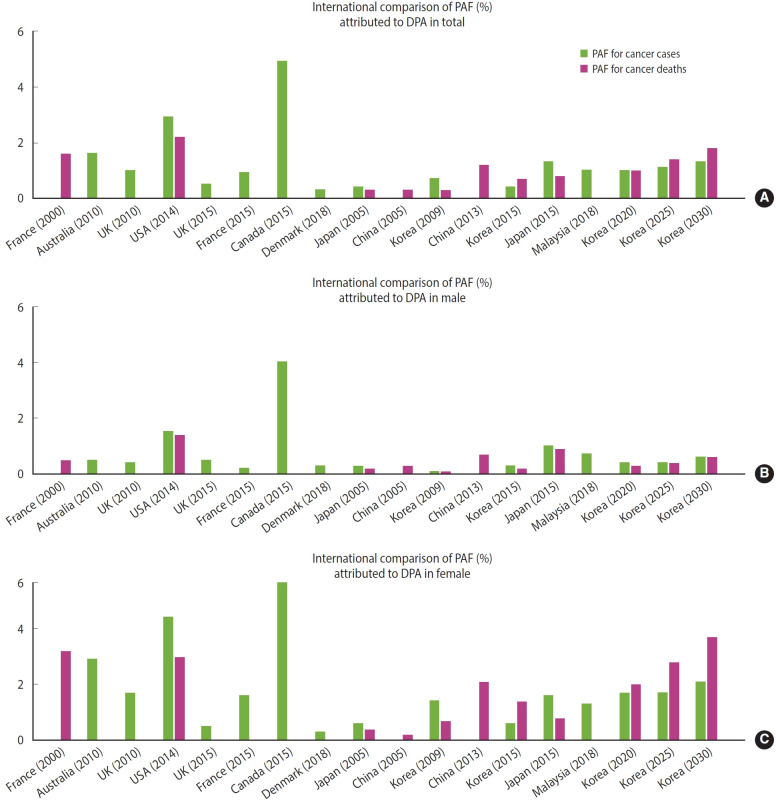
International comparison of PAF attributed to DPA (A) total, (B) male, and (C) female. PAF, population attributable fraction; DPA, deficit in physical activity.

Both France and the United Kingdom have calculated the contribution rates of PA to cancer based on the criterion of 900 METs min/wk, specifically focusing on colorectal cancer, breast cancer in postmenopausal female, and cervical cancer. These calculations yielded a contribution rate for all cancers, as previously documented [6,7]. In 2015, the contribution rates of PA to cancer incidence were 0.2% for male and 1.6% for female in France, and 0.4% for male and 1.7% for female in the United Kingdom in 2010. For the general population, the rates were 0.8% in France in 2015 and 1.0% in the United Kingdom in 2010. These results are higher than those reported for Korea in 2015. The contribution rate of PA to cancer mortality in 2000 was 0.5% for male and 4.4% for female in France, and in 2010, the rates were 1.4% for male and 3.0% for female in the United Kingdom. These values are also higher than those reported for Korea in 2015 (Figure 4) [6,7].

Western cohort studies have consistently indicated that PA has a protective effect against colorectal cancer, breast, and endometrial cancers in all female or in postmenopausal female. In contrast, Japanese cohort studies have demonstrated a preventive effect of PA on colorectal cancer in male; however, the RR for female was 1.16, indicating an increased risk and thus not supporting a preventive effect against colorectal cancer in female. Notably, although it is widely accepted that PA offers greater protection against postmenopausal breast cancer, Japanese studies have reported an RR of 0.98 for postmenopausal and 0.70 for premenopausal breast cancer [32]. This suggests a reduced risk in premenopausal female. The observed discrepancy may be due to racial differences or to factors such as higher average body mass index, increased obesity rates, and lower levels of PA in Western populations, where even minimal PA could have a significant impact.

We encountered numerous Korean cohort studies that did not provide estimates of MET-min/wk, which limited our ability to fully utilize the available cohort data. Nevertheless, we identified an exposure rate that represents DPA among Koreans. We adopted standards for appropriate weekly PA of 900, 1,260, 630, and 600 METs min/wk. Notably, the strength of our study lies in our dual approach: conducting a systematic literature review and analyzing raw data from existing Korean cohort studies. This method provided us with a comprehensive understanding of Korean cohort research. Pooling the complete set of Korean cohort data to evaluate the impact of PA on additional cancers, such as gastric cardia, bladder cancer, pancreatic cancer, ovarian cancer, kidney cancer, lung cancer, and prostate cancer, as suggested by the IARC or PAGA, would be invaluable. This would enable us to reassess the proportion of all cancers that could be attributed to PA levels.

There was a notable increase in PAF attributable to DPA in Korea in 2015 compared to 2009, affecting both sexes. Trend analysis revealed a consistent rise in the prevalence of DPA among both male and female. Although PAF was substantially higher in female than in male due to DPA, the rate of increase was more pronounced in male. Given the importance of prioritizing cancer prevention and mortality reduction strategies, focusing on this factor is crucial.

## Figures and Tables

**Figure f5-epih-47-e2025010:**
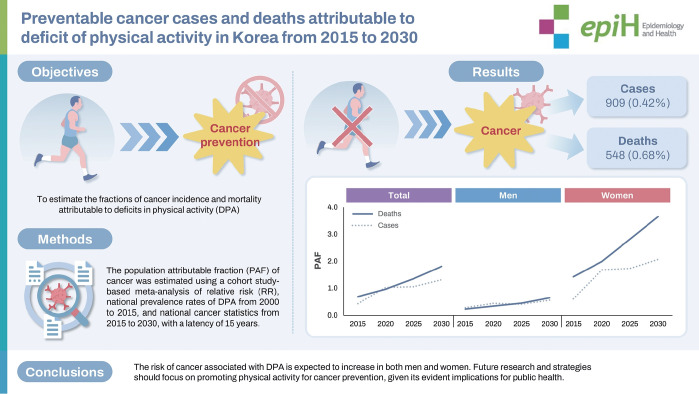


**Table 1.** PAF of DPA^1^ on cancer in Korea, 2015 to 2030

Variables	2015			2020			2025			2030		
	Observed cancer (n)	Cancer cases attributable to DPA (n)	PAF (%)	Observed cancer (n)	Cancer cases attributable to DPA (n)	PAF (%)	Observed cancer (n)	Cancer cases attributable to DPA (n)	PAF (%)	Observed cancer (n)	Cancer cases attributable to DPA (n)	PAF (%)
Cancer incidence												
Colorectal	27,120	658	2.43	35,314	1,915	5.42	35,172	2,240	6.37	43,375	3,247	7.49
Breast^2^	10,688	173	1.62	15,051	444	2.95	21,735	956	4.40	29,614	1,598	5.40
Corpus uteri	2,427	78	3.22	3,487	146	4.20	4,785	244	5.10	6,349	369	5.81
All cancer	215,570	909	0.42	246,436	2,505	1.02	327,493	3,440	1.05	397,425	5,214	1.31
Cancer death												
Colorectal	8,298	454	5.47	8,867	639	7.21	10,618	901	8.48	12,323	1,216	9.87
Breast^2^	1,701	66	3.86	2,097	100	4.78	2,581	183	7.08	3,036	262	8.63
Corpus uteri	319	29	8.99	378	47	12.30	649	95	14.70	1,081	179	16.53
All cancer	81,015	548	0.68	82,036	786	0.96	87,138	1,179	1.35	91,801	1,657	1.80

PAF, population-attributable fraction; DPA, deficit in physical activity; MET, metabolic equivalent of task.

1 DPA was defined as <900 MET-min/wk.

2 Among postmenopausal female.

**Table 2.** Population-attributable fraction of cancer attributable to DPA^1^ when using various MET criteria

Variables	Cancer incidence					Cancer mortality				
	WHO & UK criteria	WHO criterion	WHO & IPAQ criteria	French criterion	French criterion	WHO & UK criteria	WHO criterion	WHO & IPAQ criteria	French criterion	French criterion
	<900 MET-min/wk	<900 MET-min/wk with sex-specific MET calculation	<600 MET-min/wk	<1,260 MET-min/wk	<630 MET-min/wk	<900 MET-min/wk	<900 MET-min/wk with sex-specific MET calculation	<600 MET-min/wk	<1,260 MET-min/wk	<630 MET-min/wk
Total population										
Colorectal	2.43	3.98	1.88	7.20	2.08	5.47	5.38	2.54	9.64	2.81
Breast^2^	1.62	2.61	1.31	4.57	1.43	3.86	4.24	2.12	7.39	2.23
Corpus uteri	3.22	3.16	1.84	5.68	1.66	8.99	9.42	4.43	16.54	4.91
All cancer	0.42	0.67	0.32	1.20	0.35	0.68	0.68	0.32	1.21	0.35

DPA, deficit in physical activity; MET, metabolic equivalent of task; WHO, World Health Organization; IPAO, International Physical Activity Questionnaire.

1 The first criterion (900 MET-min/wk) was that based on median value of the standard of physical activity in the WHO and also used as the standard for DPA when calculating PAF in the UK; The third (600 MET-min/wk) was that based on minimum standard of physical activity suggested in the WHO and the IPAQ; The fifth was ‘minimally active’ criterion recommended by the IPAQ; The fourth and fifth criteria (<1,260 and 630 MET-min/wk) were the French criteria by which the cancer contribution of DPA was calculated.

2 For only postmenopausal female.

## Supplementary materials

Supplementary Material 1. Cancers included in the analysis of physical activity

Supplementary Material 2. Prevalence rates for the deficit in physical activity under different criteria for sensitivity analysis in Korean population

Supplementary Material 3. RR per 1 MET deficit on cancer in each cohort study and meta-analysis

Supplementary Material 4. Cohort studies1 included in the meta-analysis for the association with deficit in physical activity on the risk of specific cancer

Supplementary Material 5. Population attributable fraction (PAF) of cancer attributable to ‘deficit in physical activity’ (DPA)1 among male and female when using various MET criteria

Supplementary Material 6. Fraction (%) attributable to ‘deficit in physical activity (DPA)1’ on cancer in male and female in Korea, 2015 to 2030

Supplementary Material 7. The population attributable fraction (%) of cancer cases attributed to deficit in physical activity and proportion of specific cancers among all-cancer cases caused by deficit in physical activity in Korea, 2015.

Supplementary Material 8. The population attributable fraction (%) of cancer deaths attributed to deficit in physical activity and proportion of specific cancers among all-cancer deaths caused by deficit in physical activity in Korea, 2015.

Supplementary Material 9. The population attributable fraction (%) of cancer cases attributed to deficit in physical activity (DPA) and proportion of specific cancers among all-cancer cases caused by DPA in Korea, 2020.

Supplementary Material 10. The population attributable fraction (%) of cancer deaths attributed to deficit in physical activity (DPA) and proportion of specific cancers among all-cancer deaths caused by DPA in Korea, 2020.

Supplementary Material 11. Comparison of population attributable fraction (PAF, %) in specific cancer attributed to deficit in physical activity (DPA) when using different relative risks. WHO, World Health Organization; UK, United Kingdom; IPAQ, International Physical Activity Questionnaire.

Supplementary Material 12. Comparison of fraction (%) of deficit in physical activity on cancer in 2015 (15-latency) and in 2009 (19-latency) in Korea
