# Supplementary material for: Preventable cancer cases and deaths attributable to deficit of physical activity in Korea from 2015 to 2030
Source: Epidemiol Health. 2025 Jan 27;47:e2025010. doi: 10.4178/epih.e2025010 (PMC12531471; doi:10.4178/epih.e2025010)
Supplement: Supplementary Material 1. — Cancers included in the analysis of physical activity [file epih-47-e2025010-Supplementary-1.docx]

Supplementary Material 1. Cancers included in the analysis of physical activity

| **Factor** | **Selected cancers^1^** | **Criteria** |
| --- | --- | --- |
| Physical activity^1^ | Colorectal (C18-20) | WCRF-Convincing  WCRF-Probable |
|  | Breast (postmenopausal) (C50)  Corpus uteri (C54) ^2^ |  |
| Vigorous physical activity ^2^ | Breast (postmenopausal) (C50) | WCRF-Probable |

Abbreviation: WCRF, World Cancer Research Fund.

1. All physical activity, including vigorous physical activity.

Any types of physical activity (including vigorous physical activity) is decreased at risk of postmenopausal breast cancer but vigorous physical activity is decreased at risk of premenopausal breast cancer.

2. The reason we used "corpus uteri cancer" in our study instead of "endometrial cancer" is because the distinction between these two types of cancer is often minimal in clinical practice and epidemiological studies. Endometrial cancer is the most common form of corpus uteri cancer, accounting for over 90% of cases. Given that both terms refer to cancers of the uterine body, we chose to use "corpus uteri cancer" to encompass all relevant cases, including both endometrial cancer and other rare types of uterine cancer, while aligning with the terminology commonly used in broader epidemiological research.
