# Supplementary material for: Preventable cancer cases and deaths attributable to deficit of physical activity in Korea from 2015 to 2030
Source: Epidemiol Health. 2025 Jan 27;47:e2025010. doi: 10.4178/epih.e2025010 (PMC12531471; doi:10.4178/epih.e2025010)
Supplement: Supplementary Material 2. — Prevalence rates for the deficit in physical activity under different criteria for sensitivity analysis in Korean population [file epih-47-e2025010-Supplementary-2.docx]

Supplementary Material 2. Prevalence rates for the deficit in physical activity under different criteria for sensitivity analysis in Korean population

|  |  | **2000^1^** | | **2005^2^** | | **2010** | | **2015** | |
| --- | --- | --- | --- | --- | --- | --- | --- | --- | --- |
|  | **Criteria of ‘the deficit in physical activity’** | **Prevalence**  **rates**  **(%)** | **Mean DPA (MET min**  **/week)^2^** | **Prevalence**  **rates**  **(%)** | **Mean DPA (MET min**  **/week)^2^** | **Prevalence**  **rates**  **(%)** | **Mean DPA (MET min**  **/week)^2^** | **Prevalence**  **rates**  **(%)** | **Mean DPA (MET min**  **/week)^2^** |
| **Male** |  |  |  |  |  |  |  |  |  |
| Non-specific | <900 MET min/week | 23.55 | 8.66 | 31.80 | 9.26 | 36.01 | 9.57 | 45.57 | 10.01 |
| MET levels | <600 MET min/week | 18.00 | 5.40 | 22.99 | 6.43 | 27.70 | 6.41 | 35.53 | 6.85 |
|  | <1,260 MET min/week | 34.85 | 11.07 | 37.29 | 13.62 | 43.55 | 13.52 | 54.55 | 14.26 |
|  | <630 MET min/week | 18.69 | 5.70 | 23.57 | 6.78 | 28.33 | 6.79 | 36.70 | 7.11 |
| Sex-specific | <900 MET min/week | 23.21 | 8.67 | 31.56 | 9.20 | 35.89 | 9.50 | 45.20 | 10.00 |
|  |  |  |  |  |  |  |  |  |  |
| **Female** |  |  |  |  |  |  |  |  |  |
| Non-specific | <900 MET min/week | 28.47 | 9.16 | 36.48 | 9.40 | 43.29 | 9.69 | 49.55 | 9.72 |
| MET levels | <600 MET min/week | 23.15 | 5.27 | 27.70 | 6.23 | 32.00 | 6.65 | 38.20 | 6.46 |
|  | <1,260 MET min/week | 39.46 | 11.77 | 42.58 | 13.87 | 49.05 | 14.12 | 58.31 | 14.02 |
|  | <630 MET min/week | 23.56 | 5.73 | 28.19 | 6.67 | 33.03 | 7.01 | 39.64 | 6.83 |
| Sex-specific | <900 MET min/week | 28.46 | 9.00 | 37.08 | 9.23 | 39.14 | 9.58 | 49.73 | 9.61 |

### Abbreviations: DPA, deficit in physical activity; MET, Metabolic equivalent of task.

### 1. The prevalence rates of the DPA in 2000 and 2005 were estimated using the Korea National Health and Nutrition Examination Survey (KNHANES) data in 2005 and 2008, since it was difficult to estimate the MET in 1998 and 2001 questionnaires.

### 2. Mean DPA indicates average degree of physical activity less than criteria.
