# Supplementary material for: Preventable cancer cases and deaths attributable to deficit of physical activity in Korea from 2015 to 2030
Source: Epidemiol Health. 2025 Jan 27;47:e2025010. doi: 10.4178/epih.e2025010 (PMC12531471; doi:10.4178/epih.e2025010)
Supplement: Supplementary Material 3. — RR per 1 MET deficit on cancer in each cohort study and meta-analysis [file epih-47-e2025010-Supplementary-3.docx]

Supplementary Material 3. RR per 1 MET deficit on cancer in each cohort study and meta-analysis

|  |  | **Variable** | **Study** | **RR (95% CI)**  **per 1 MET deficit** | | |
| --- | --- | --- | --- | --- | --- | --- |
| **Incidence** |  |  |  |  |  |  |
| Colorectal | Male | Total physical activity | Meta-analysis | 1.009 | 0.992 | 1.027 |
| Colorectal | Female | Total physical activity | Meta-analysis | 1.012 | 0.968 | 1.058 |
| Breast | Female | Total physical activity | Meta-analysis | 1.006 | 0.975 | 1.038 |
| Breast | Female | Vigorous physical activity | Meta-analysis | 1.055 | 0.773 | 1.724 |
| Corpus uteri | Female | Total physical activity | Meta-analysis | 1.012 | 0.942 | 1.087 |
|  |  |  |  |  |  |  |
| **Death** |  |  |  |  |  |  |
| Colorectal | Male | Total physical activity | Meta-analysis | 1.011 | 0.989 | 1.033 |
| Colorectal | Female | Total physical activity | Meta-analysis | 1.035 | 1.004 | 1.067 |
| Breast | Female | Total physical activity | Meta-analysis | 1.014 | 0.975 | 1.055 |
| Breast | Female | Vigorous physical activity | Meta-analysis | 1.009 | 0.782 | 1.302 |
| Corpus uteri | Female | Total physical activity | Meta-analysis | 1.033 | 0.947 | 1.129 |

Abbreviation: RR, Relative risk; MET, Metabolic equivalent of task.

In PAF calculation, the RR for breast cancer death due to vigorous physical activity was replaced to the RR for breast cancer incidence due to wide confidence interval.
