# Supplementary material for: Preventable cancer cases and deaths attributable to deficit of physical activity in Korea from 2015 to 2030
Source: Epidemiol Health. 2025 Jan 27;47:e2025010. doi: 10.4178/epih.e2025010 (PMC12531471; doi:10.4178/epih.e2025010)
Supplement: Supplementary Material 4. — Cohort studies1 included in the meta-analysis for the association with deficit in physical activity on the risk of specific cancer [file epih-47-e2025010-Supplementary-4.docx]

Supplementary Material 4. Cohort studies^1^ included in the meta-analysis for the association with deficit in physical activity on the risk of specific cancer

| **Colorectal cancer** | **Corus uterine cancer** |
| --- | --- |
| Ballard-barbash et al. (1990) | Friberg et al. (2006) |
| Wannamethee et al. (2001) | Schouten et al. (2004) |
| Nilsen et al. (2002) | Patel et al. (2008) |
| Larsson et al. (2006) |  |
| Lee et al. (2007) |  |
| Mok et al. (2016) |  |
| KMCC | KMCC |
| H-PEACE | NWS/DGS |
| NWS/DGS |  |
| **Breast cancer** |  |
| Howard et al. (2009) |  |
| Hildebrand et al. (2013) |  |
| Fournier et al. (2014) |  |
| Rosenberg et al. (2014) |  |
| Dallal et al. (2014) |  |
| Silvera et al. (2006) |  |
| Lee et al. (2001) |  |
| Rockhill et al. (1999) |  |
| Hållmarker et al. (2015) |  |
| Boeke et al. (2014) |  |
| Catsburg et al. (2014) |  |
| Steindorf et al. (2013) |  |
| Breslow et al. (2001) |  |
| Sesso et al. (1998) |  |
| Thune et al. (1997) |  |
| Suzuki et al. (2011) |  |
| KMCC |  |
| KoGES |  |
| H-PEACE |  |
| NWS/DGS |  |

Abbreviation: KMCC, Korea Multicenter Cancer Cohort study; NWS/DGS, Namwon-Donggu study; H-PEACE, Health and Prevention Enhancement study; KoGES, Korea Genomic and Epidemiologic Study; KNHANES, Korea National Health And Nutritional Examination Survey-based Cohort. All cohort studies were described in prior paper (Lee S et al. J Prev Med Public Health 2022)

1. We wanted to calculate PAFs in 2015 and 2020 using the prevalence rates of deficit in physical activity in 2000 and 2005 (15-latency), respectively. Therefore, the literature selected in the systematic review was based on cohort studies from 1990 to 2019 that presented RR values for cancer risk attributed to deficit in physical activity.

**[Reference for meta-analysis]**

1. Ballard-Barbash R, Schatzkin A, Albanes D, Schiffman MH, Kreger BE, Kannel WB, et al. Physical activity and risk of large bowel cancer in the Framingham Study. Cancer Res. 1990;50(12):3610-3.

2. Wannamethee SG, Shaper AG, Walker M. Physical activity and risk of cancer in middle-aged men. Br J Cancer. 2001;85(9):1311-6.

3. Nilsen TI, Vatten LJ. Prospective study of colorectal cancer risk and physical activity, diabetes, blood glucose and BMI: exploring the hyperinsulinaemia hypothesis. Br J Cancer. 2001;84(3):417-22.

4. Larsson SC, Rutegård J, Bergkvist L, Wolk A. Physical activity, obesity, and risk of colon and rectal cancer in a cohort of Swedish men. Eur J Cancer. 2006;42(15):2590-7.

5. Lee KJ, Inoue M, Otani T, Iwasaki M, Sasazuki S, Tsugane S. Physical activity and risk of colorectal cancer in Japanese men and women: the Japan Public Health Center-based prospective study. Cancer Causes Control. 2007;18(2):199-209.

6. Friberg E, Mantzoros CS, Wolk A. Physical activity and risk of endometrial cancer: a population-based prospective cohort study. Cancer Epidemiol Biomarkers Prev. 2006;15(11):2136-40.

7. Schouten LJ, Goldbohm RA, van den Brandt PA. Anthropometry, physical activity, and endometrial cancer risk: results from the Netherlands Cohort Study. J Natl Cancer Inst. 2004;96(21):1635-8.

8. Patel AV, Feigelson HS, Talbot JT, McCullough ML, Rodriguez C, Patel RC, et al. The role of body weight in the relationship between physical activity and endometrial cancer: results from a large cohort of US women. Int J Cancer. 2008;123(8): 1877-82.

9. Howard RA, Leitzmann MF, Linet MS, Freedman DM. Physical activity and breast cancer risk among pre- and postmenopausal women in the U.S. Radiologic Technologists cohort. Cancer Causes Control. 2009;20(3):323-33.

10. Hildebrand JS, Gapstur SM, Campbell PT, Gaudet MM, Patel AV. Recreational physical activity and leisure-time sitting in relation to postmenopausal breast cancer risk. Cancer Epidemiol Biomarkers Prev. 2013;22(10):1906-12.

11. Fournier A, Dos Santos G, Guillas G, Bertsch J, Duclos M, Boutron-Ruault MC, et al. Recent recreational physical activity and breast cancer risk in postmenopausal women in the E3N cohort. Cancer Epidemiol Biomarkers Prev. 2014;23(9):1893-902.

12. Rosenberg L, Palmer JR, Bethea TN, Ban Y, Kipping-Ruane K, Adams-Campbell LL. A prospective study of physical activity and breast cancer incidence in African-American women. Cancer Epidemiol Biomarkers Prev. 2014;23(11):2522-31.

13. Dallal CM, Sullivan-Halley J, Ross RK, Wang Y, Deapen D, Horn-Ross PL, et al. Long-term recreational physical activity and risk of invasive and in situ breast cancer: the California teachers study. Arch Intern Med. 2007;167(4):408-15.

14. Silvera SA, Jain M, Howe GR, Miller AB, Rohan TE. Energy balance and breast cancer risk: a prospective cohort study. Breast Cancer Res Treat. 2006;97(1):97-106.

15. Lee IM, Rexrode KM, Cook NR, Hennekens CH, Burin JE. Physical activity and breast cancer risk: the Women's Health Study (United States). Cancer Causes Control. 2001;12(2):137-45.

16. Rockhill B, Willett WC, Hunter DJ, Manson JE, Hankinson SE, Colditz GA. A prospective study of recreational physical activity and breast cancer risk. Arch Intern Med. 1999;159(19):2290-6.

17. Hållmarker U, James S, Michaëlsson K, Ärnlöv J, Sandin F, Holmberg L. Cancer incidence in participants in a long-distance ski race (Vasaloppet, Sweden) compared to the background population. Eur J Cancer. 2015;51(4):558-68.

18. Boeke CE, Eliassen AH, Oh H, Spiegelman D, Willett WC, Tamimi RM. Adolescent physical activity in relation to breast cancer risk. Breast Cancer Res Treat. 2014;145(3):715-24.

19. Catsburg C, Kirsh VA, Soskolne CL, Kreiger N, Bruce E, Ho T, et al. Associations between anthropometric characteristics, physical activity, and breast cancer risk in a Canadian cohort. Breast Cancer Res Treat. 2014;145(2):545-52.

20. Steindorf K, Ritte R, Eomois PP, Lukanova A, Tjonneland A, Johnsen NF, et al. Physical activity and risk of breast cancer overall and by hormone receptor status: the European prospective investigation into cancer and nutrition. Int J Cancer. 2013;132(7):1667-78.

21. Breslow RA, Ballard-Barbash R, Munoz K, Graubard BI. Long-term recreational physical activity and breast cancer in the National Health and Nutrition Examination Survey I epidemiologic follow-up study. Cancer Epidemiol Biomarkers Prev. 2001;10(7):805-8.

22. Sesso HD, Paffenbarger RS, Jr., Lee IM. Physical activity and breast cancer risk in the College Alumni Health Study (United States). Cancer Causes Control. 1998;9(4):433-9.

23. Thune I, Brenn T, Lund E, Gaard M. Physical activity and the risk of breast cancer. N Engl J Med. 1997;336(18):1269-75.

24. Suzuki R, Iwasaki M, Yamamoto S, Inoue M, Sasazuki S, Sawada N, et al. Leisure-time physical activity and breast cancer risk defined by estrogen and progesterone receptor status--the Japan Public Health Center-based Prospective Study. Prev Med. 2011;52(3-4):227-33.
