# Supplementary material for: Preventable cancer cases and deaths attributable to deficit of physical activity in Korea from 2015 to 2030
Source: Epidemiol Health. 2025 Jan 27;47:e2025010. doi: 10.4178/epih.e2025010 (PMC12531471; doi:10.4178/epih.e2025010)
Supplement: Supplementary Material 5. — Population attributable fraction (PAF) of cancer attributable to ‘deficit in physical activity’ (DPA)1 among male and female when using various MET criteria [file epih-47-e2025010-Supplementary-5.docx]

Supplementary Material 5. Population attributable fraction (PAF) of cancer attributable to ‘deficit in physical activity’ (DPA)^1^ among male and female when using various MET criteria

|  | **Cancer incidence** | | | | | **Cancer mortality** | | | | |
| --- | --- | --- | --- | --- | --- | --- | --- | --- | --- | --- |
|  | **WHO & UK criteria** | **WHO**  **criterion** | **WHO & IPAQ**  **criteria** | **French**  **criterion** | **French**  **criterion** | **WHO & UK criteria** | **WHO**  **criterion** | **WHO & IPAQ**  **criteria** | **French**  **criterion** | **French**  **criterion** |
|  | **<900 MET minute/ week** | **<900 MET minute/week**  **with sex-specific**  **MET calculation** | **<600 MET minute/week** | **<1,260 MET minute/ week** | **<630 MET minute/week** | **<900 MET minute/ week** | **<900 MET minute/week**  **with sex-specific**  **MET calculation** | **<600 MET minute/week** | **<1,260 MET minute/ week** | **<630 MET minute/week** |
| **Male** |  |  |  |  |  |  |  |  |  |  |
| Colorectal | 1.88 | 1.86 | 0.89 | 3.54 | 0.98 | 2.31 | 2.28 | 1.09 | 4.34 | 1.20 |
| Breast^1^ |  |  |  |  |  |  |  |  |  |  |
| Corpus uteri |  |  |  |  |  |  |  |  |  |  |
| **All cancer** | **0.27** | **0.26** | **0.13** | **0.50** | **0.14** | **0.22** | **0.21** | **0.10** | **0.41** | **0.11** |
| **Female** |  |  |  |  |  |  |  |  |  |  |
| Colorectal | 3.22 | 7.08 | 3.33 | 12.54 | 3.69 | 9.60 | 9.42 | 4.43 | 16.54 | 4.91 |
| Breast^1^ | 1.62 | 2.61 | 1.31 | 4.57 | 1.43 | 3.86 | 4.24 | 2.12 | 7.39 | 2.23 |
| Corpus uteri | 3.22 | 3.16 | 1.84 | 5.68 | 1.66 | 8.99 | 9.42 | 4.43 | 16.54 | 4.91 |
| **All cancer** | **0.60** | **1.11** | **0.54** | **1.97** | **0.59** | **1.42** | **1.43** | **0.68** | **2.51** | **0.35** |

Abbreviation: PAF, Population attributable fraction; DPA, Deficit in physical activity.

1. WHO recommended 150-300 minutes of moderated activity hour per week. When the median MET of moderate activities put ‘4 MET’, the standard of PA in the WHO is ‘600-1200 MET minutes per week. The 1st criterion (900 MET minute/week) was that based on median value of the standard of physical activity in the WHO and also used as the standard for DPA when calculating PAF in the UK. The 3rd (600 MET minute/week) was that based on minimum standard of physical activity suggested in the WHO and the IPAQ. The 5th was ‘minimally active’ criterion recommended by the IPAQ. The 4th and 5th criteria (<1,260 MET minute/week and 630 MET minute/week) were the French criteria by which the cancer contribution of DPA was calculated.

2. Among postmenopausal female.
