# Supplementary material for: Preventable cancer cases and deaths attributable to deficit of physical activity in Korea from 2015 to 2030
Source: Epidemiol Health. 2025 Jan 27;47:e2025010. doi: 10.4178/epih.e2025010 (PMC12531471; doi:10.4178/epih.e2025010)
Supplement: Supplementary Material 6. — Fraction (%) attributable to ‘deficit in physical activity (DPA)1’ on cancer in male and female in Korea, 2015 to 2030 [file epih-47-e2025010-Supplementary-6.docx]

Supplementary Material 6. Fraction (%) attributable to ‘deficit in physical activity (DPA)^1^’ on cancer in male and female in Korea, 2015 to 2030

|  | **2015** | | | **2020** | | | **2025** | | | **2030** | | |
| --- | --- | --- | --- | --- | --- | --- | --- | --- | --- | --- | --- | --- |
|  | **Observed**  **Cancer**  **N** | **Attributable**  **Cancer by DPA**  **N** | **PAF**  **%** | **Observed**  **Cancer**  **N** | **Attributable**  **Cancer by DPA**  **N** | **PAF**  **%** | **Observed**  **Cancer**  **N** | **Attributable**  **Cancer by DPA**  **N** | **PAF**  **%** | **Observed**  **Cancer**  **N** | **Attributable**  **Cancer by DPA**  **N** | **PAF**  **%** |
| **Male** |  |  |  |  |  |  |  |  |  |  |  |  |
| **Cancer Incidence** |  |  |  |  |  |  |  |  |  |  |  |  |
| Colorectal | 16,103 | 303 | 1.88 | 20,761 | 561 | 2.70 | 21,079 | 664 | 3.15 | 26,287 | 1,087 | 4.14 |
| Breast^2^ |  |  |  |  |  |  |  |  |  |  |  |  |
| Corpus uteri |  |  |  |  |  |  |  |  |  |  |  |  |
| **All cancer** | **113,739** | **303** | **0.27** | **129,839** | **561** | **0.43** | **166,455** | **664** | **0.40** | **196,843** | **1,087** | **0.55** |
| **Cancer death** |  |  |  |  |  |  |  |  |  |  |  |  |
| Colorectal | 4,698 | 109 | 2.31 | 5,027 | 167 | 3.32 | 6,091 | 235 | 3.86 | 7,163 | 363 | 5.06 |
| Breast^2^ |  |  |  |  |  |  |  |  |  |  |  |  |
| Corpus uteri |  |  |  |  |  |  |  |  |  |  |  |  |
| **All cancer** | **50,149** | **109** | **0.22** | **50,705** | **167** | **0.33** | **53,589** | **235** | **0.44** | **56,388** | **363** | **0.64** |
| **Female** |  |  |  |  |  |  |  |  |  |  |  |  |
| **Cancer Incidence** |  |  |  |  |  |  |  |  |  |  |  |  |
| Colorectal | 11,017 | 355 | 3.22 | 14,553 | 1,354 | 9.30 | 14,093 | 1,576 | 11.18 | 17,088 | 2,159 | 12.64 |
| Breast^2^ | 10,688 | 173 | 1.62 | 15,051 | 444 | 2.95 | 21,735 | 957 | 4.40 | 29,614 | 1,598 | 5.40 |
| Corpus uteri | 2,427 | 78 | 3.22 | 3,487 | 147 | 4.20 | 4,785 | 244 | 5.10 | 6,349 | 369 | 5.81 |
| **All cancer** | **101,831** | **606** | **0.60** | **116,597** | **1,944** | **1.67** | **161,038** | **2,776** | **1.72** | **200,582** | **4,127** | **2.06** |
| **Cancer death** |  |  |  |  |  |  |  |  |  |  |  |  |
| Colorectal | 3,600 | 345 | 9.60 | 3,840 | 472 | 12.30 | 4,527 | 666 | 14.70 | 5,160 | 853 | 16.53 |
| Breast^2^ | 1,701 | 66 | 3.86 | 2,097 | 100 | 4.78 | 2,581 | 183 | 7.08 | 3,036 | 262 | 8.63 |
| Corpus uteri | 319 | 29 | 8.99 | 378 | 46 | 12.30 | 649 | 95 | 14.70 | 1,081 | 179 | 16.53 |
| **All cancer** | **30,866** | **440** | **1.42** | **31,331** | **619** | **1.98** | **33,549** | **944** | **2.81** | **35,413** | **1,294** | **3.65** |

Abbreviation: PAF, Population attributable fraction; DPA, Deficit in physical activity.

1. The DPA was set as ‘<900 METs minute/week’.

2. Among postmenopausal female.
