# Supplementary material for: Preventable cancer cases and deaths attributable to deficit of physical activity in Korea from 2015 to 2030
Source: Epidemiol Health. 2025 Jan 27;47:e2025010. doi: 10.4178/epih.e2025010 (PMC12531471; doi:10.4178/epih.e2025010)
Supplement: Supplementary Material 7. — The population attributable fraction (%) of cancer cases attributed to deficit in physical activity and proportion of specific cancers among all-cancer cases caused by deficit in physical activity in Korea, 2015. [file epih-47-e2025010-Supplementary-7.pptx]

## Slide 1
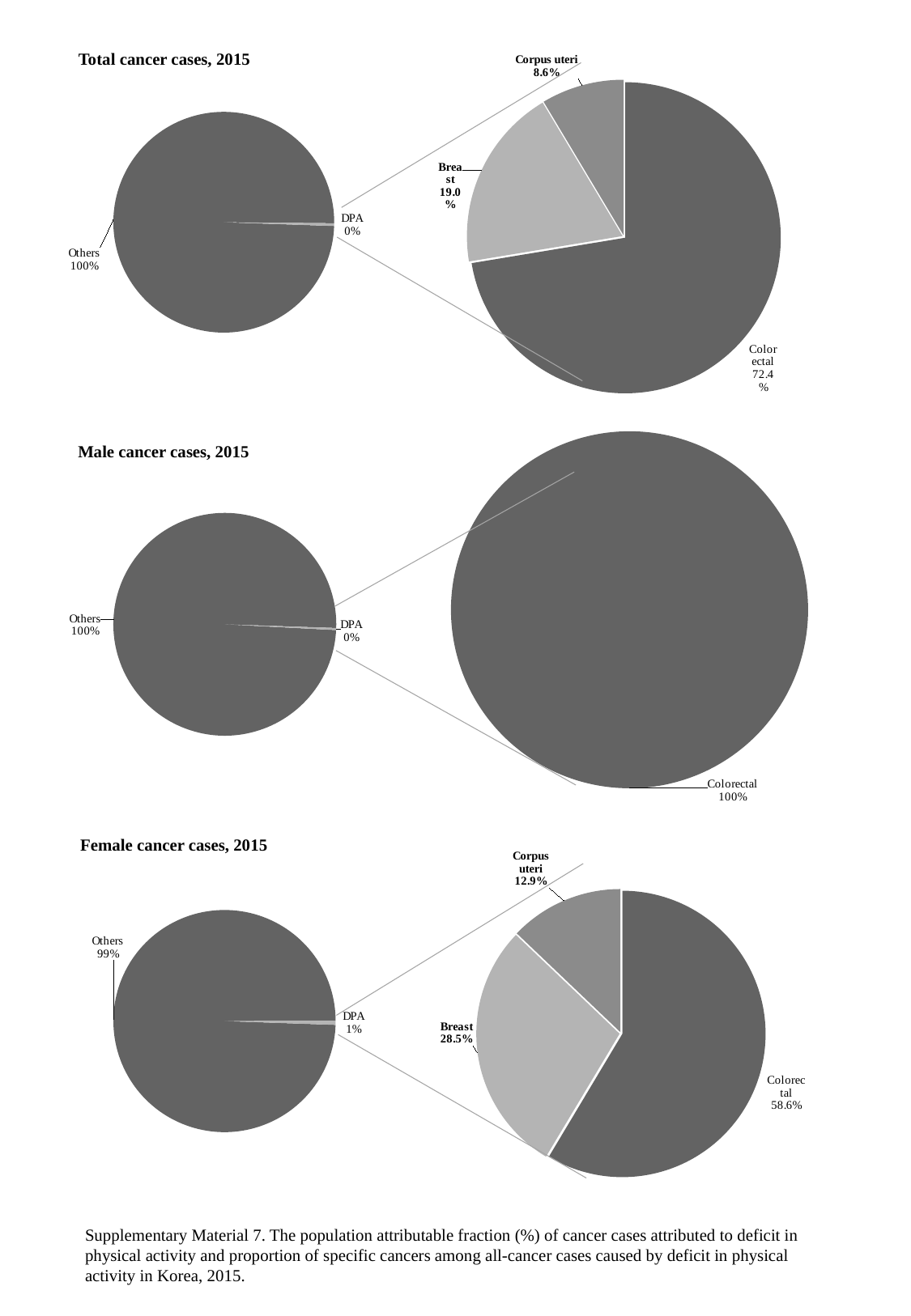

### Chart
| Category | |
|---|---|
| Colorectal | 658.0 |
| Breast | 173.0 |
| Corpus uteri | 78.0 |
### Chart
| Category | |
|---|---|
| Others | 99.6 |
| DPA | 0.4 |Total cancer cases, 2015
### Chart
| Category | |
|---|---|
| Colorectal | 303.0 |
### Chart
| Category | |
|---|---|
| Others | 99.7 |
| DPA | 0.3 |Male cancer cases, 2015
### Chart
| Category | |
|---|---|
| Colorectal | 355.0 |
| Breast | 173.0 |
| Corpus uteri | 78.0 |
### Chart
| Category | |
|---|---|
| Others | 99.4 |
| DPA | 0.6 |Female cancer cases, 2015
Supplementary Material 7. The population attributable fraction (%) of cancer cases attributed to deficit in physical activity and proportion of specific cancers among all-cancer cases caused by deficit in physical activity in Korea, 2015.
