# Supplementary material for: Preventable cancer cases and deaths attributable to deficit of physical activity in Korea from 2015 to 2030
Source: Epidemiol Health. 2025 Jan 27;47:e2025010. doi: 10.4178/epih.e2025010 (PMC12531471; doi:10.4178/epih.e2025010)
Supplement: Supplementary Material 8. — The population attributable fraction (%) of cancer deaths attributed to deficit in physical activity and proportion of specific cancers among all-cancer deaths caused by deficit in physical activity in Korea, 2015. [file epih-47-e2025010-Supplementary-8.pptx]

## Slide 1
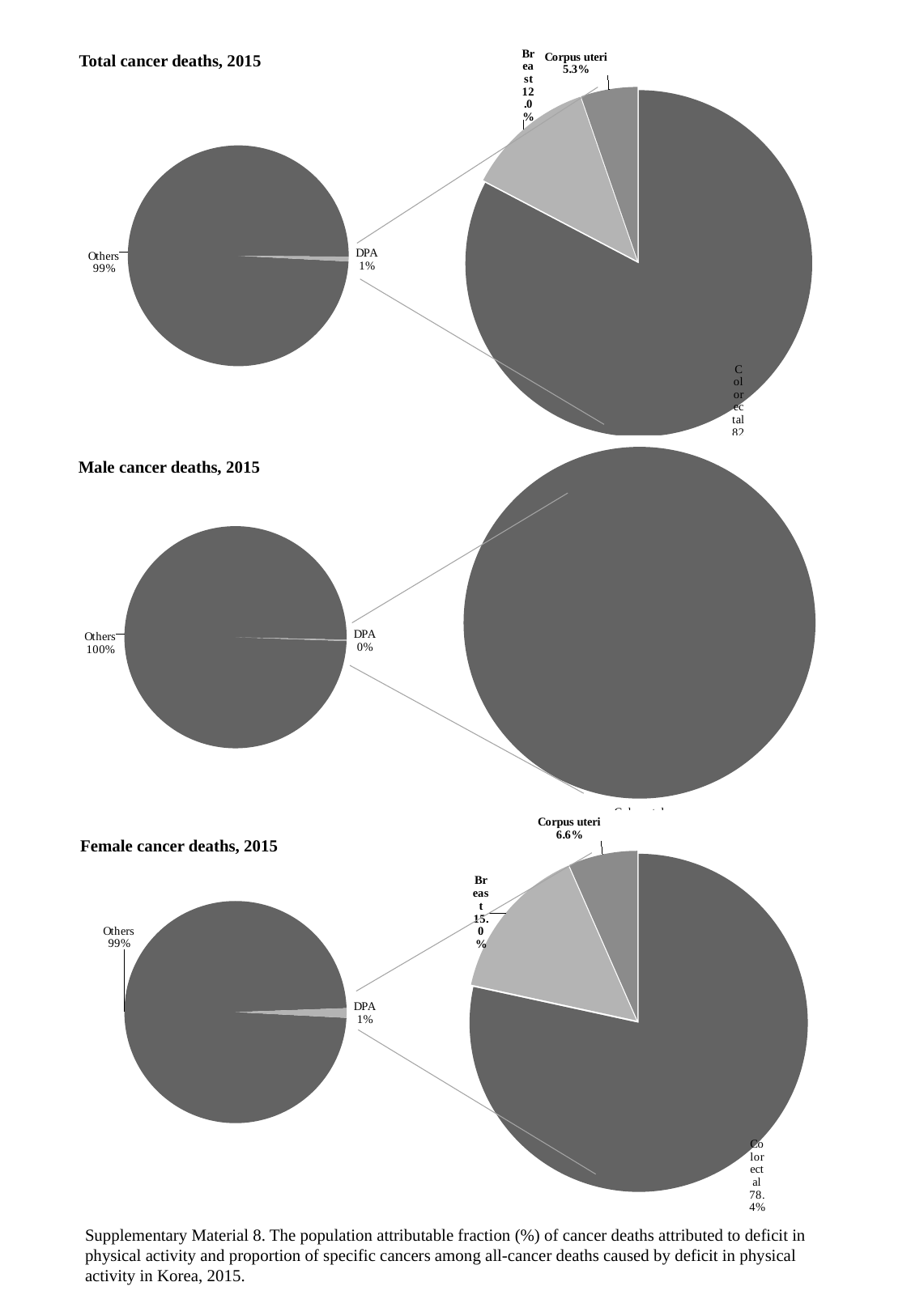

Total cancer deaths, 2015
### Chart
| Category | |
|---|---|
| Colorectal | 454.0 |
| Breast | 66.0 |
| Corpus uteri | 29.0 |
### Chart
| Category | |
|---|---|
| Others | 99.3 |
| DPA | 0.7 |
### Chart
| Category | |
|---|---|
| Colorectal | 109.0 |
### Chart
| Category | |
|---|---|
| Others | 99.8 |
| DPA | 0.2 |Male cancer deaths, 2015
### Chart
| Category | |
|---|---|
| Colorectal | 345.0 |
| Breast | 66.0 |
| Corpus uteri | 29.0 |
### Chart
| Category | |
|---|---|
| Others | 98.6 |
| DPA | 1.4 |Female cancer deaths, 2015
Supplementary Material 8. The population attributable fraction (%) of cancer deaths attributed to deficit in physical activity and proportion of specific cancers among all-cancer deaths caused by deficit in physical activity in Korea, 2015.
