# Supplementary material for: Preventable cancer cases and deaths attributable to deficit of physical activity in Korea from 2015 to 2030
Source: Epidemiol Health. 2025 Jan 27;47:e2025010. doi: 10.4178/epih.e2025010 (PMC12531471; doi:10.4178/epih.e2025010)
Supplement: Supplementary Material 9. — The population attributable fraction (%) of cancer cases attributed to deficit in physical activity (DPA) and proportion of specific cancers among all-cancer cases caused by DPA in Korea, 2020. [file epih-47-e2025010-Supplementary-9.pptx]

## Slide 1
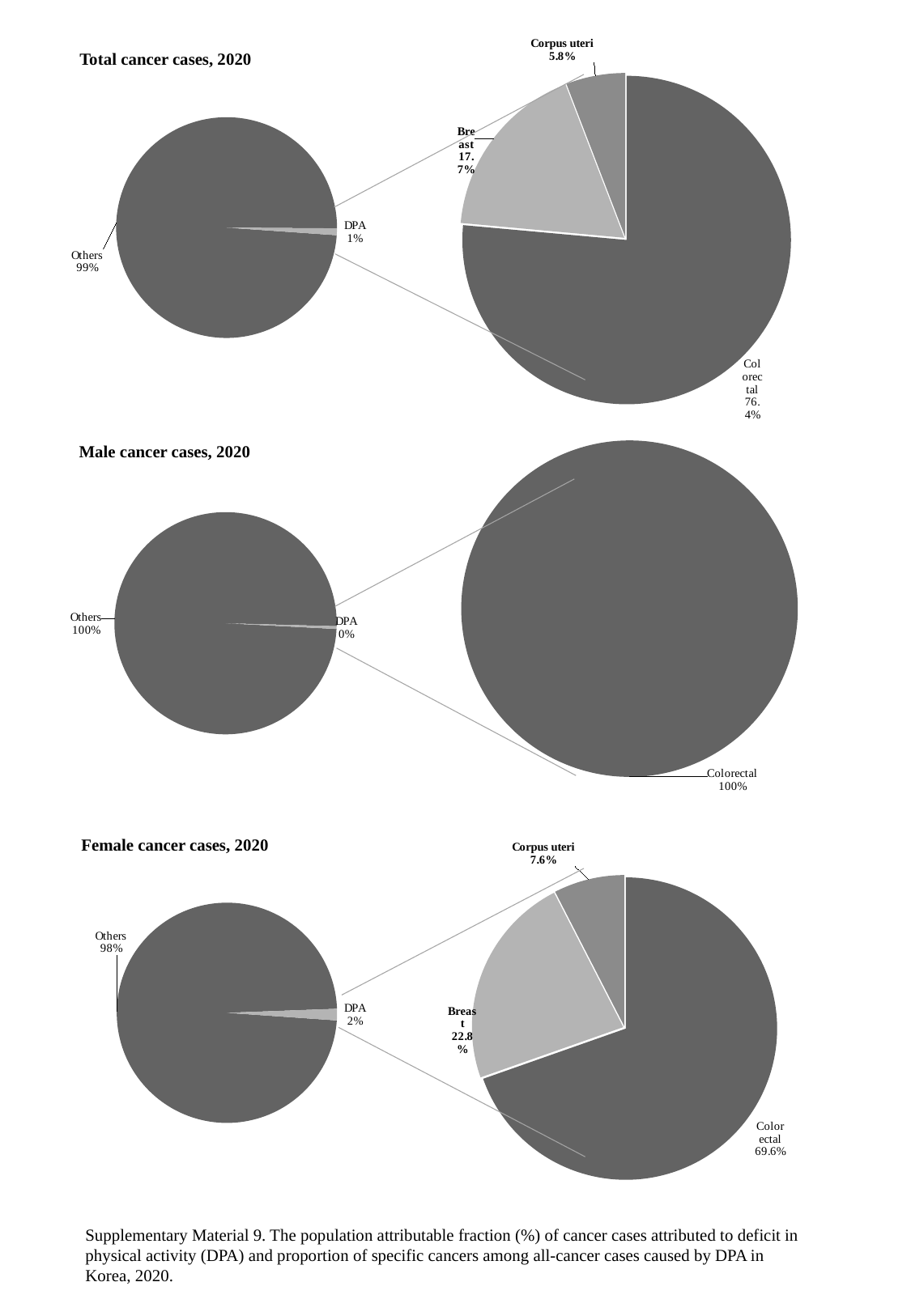

### Chart
| Category | |
|---|---|
| Colorectal | 1915.0 |
| Breast | 444.0 |
| Corpus uteri | 146.0 |
### Chart
| Category | |
|---|---|
| Others | 99.0 |
| Smoking | 1.0 |Total cancer cases, 2020
### Chart
| Category | |
|---|---|
| Colorectal | 561.0 |
### Chart
| Category | |
|---|---|
| Others | 99.6 |
| Smoking | 0.4 |Male cancer cases, 2020
### Chart
| Category | |
|---|---|
| Colorectal | 1354.0 |
| Breast | 444.0 |
| Corpus uteri | 147.0 |
### Chart
| Category | |
|---|---|
| Others | 98.3 |
| Smoking | 1.7 |Female cancer cases, 2020
Supplementary Material 9. The population attributable fraction (%) of cancer cases attributed to deficit in physical activity (DPA) and proportion of specific cancers among all-cancer cases caused by DPA in Korea, 2020.
