# Supplementary material for: Preventable cancer cases and deaths attributable to deficit of physical activity in Korea from 2015 to 2030
Source: Epidemiol Health. 2025 Jan 27;47:e2025010. doi: 10.4178/epih.e2025010 (PMC12531471; doi:10.4178/epih.e2025010)
Supplement: Supplementary Material 10. — The population attributable fraction (%) of cancer deaths attributed to deficit in physical activity (DPA) and proportion of specific cancers among all-cancer deaths caused by DPA in Korea, 2020. [file epih-47-e2025010-Supplementary-10.pptx]

## Slide 1
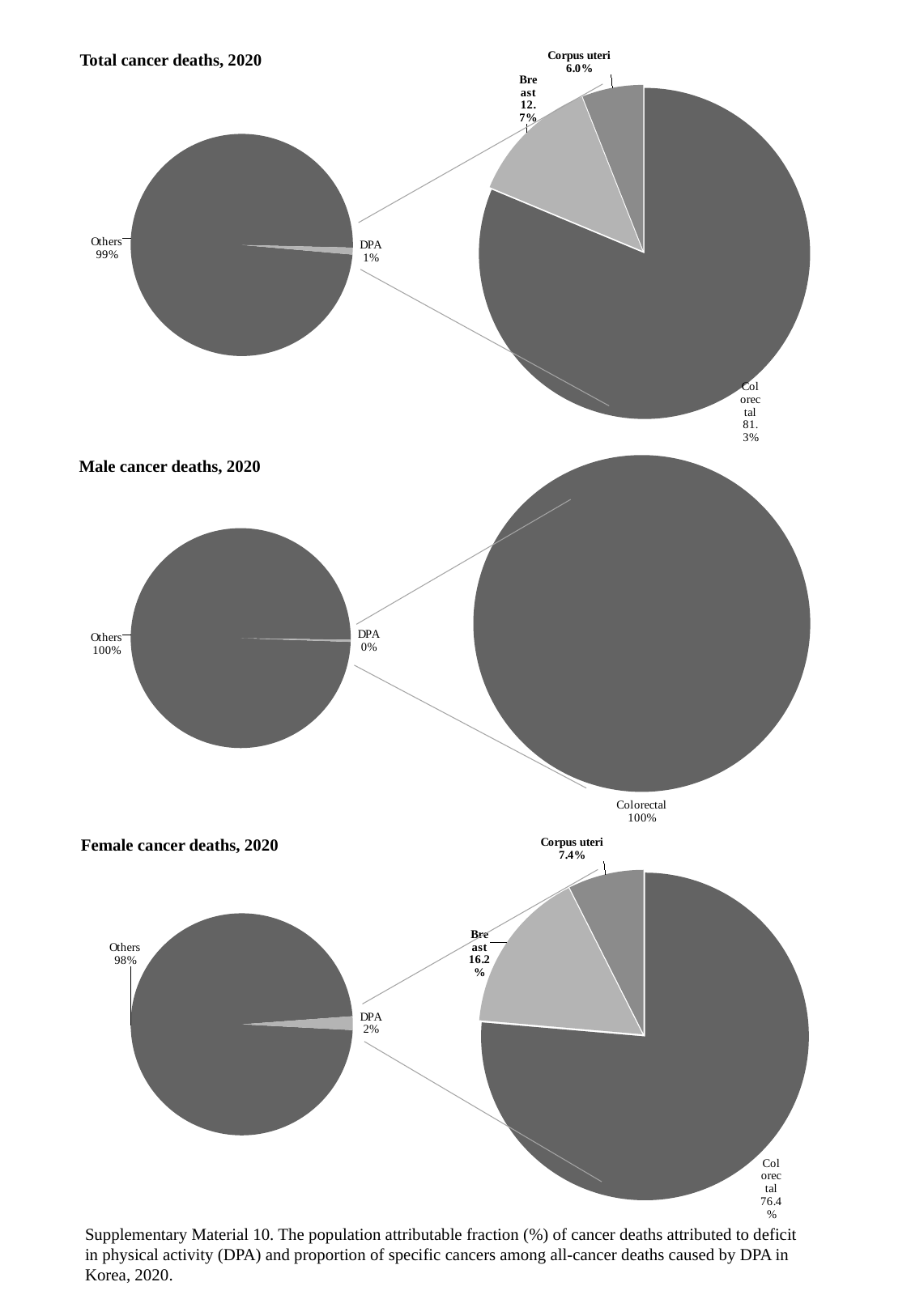

Total cancer deaths, 2020
### Chart
| Category | |
|---|---|
| Colorectal | 639.0 |
| Breast | 100.0 |
| Corpus uteri | 47.0 |
### Chart
| Category | |
|---|---|
| Others | 99.0 |
| Smoking | 1.0 |
### Chart
| Category | |
|---|---|
| Colorectal | 167.0 |
### Chart
| Category | |
|---|---|
| Others | 99.7 |
| Smoking | 0.3 |Male cancer deaths, 2020
### Chart
| Category | |
|---|---|
| Colorectal | 472.0 |
| Breast | 100.0 |
| Corpus uteri | 46.0 |
### Chart
| Category | |
|---|---|
| Others | 98.0 |
| Smoking | 2.0 |Female cancer deaths, 2020
Supplementary Material 10. The population attributable fraction (%) of cancer deaths attributed to deficit in physical activity (DPA) and proportion of specific cancers among all-cancer deaths caused by DPA in Korea, 2020.
