# Supplementary material for: Preventable cancer cases and deaths attributable to deficit of physical activity in Korea from 2015 to 2030
Source: Epidemiol Health. 2025 Jan 27;47:e2025010. doi: 10.4178/epih.e2025010 (PMC12531471; doi:10.4178/epih.e2025010)
Supplement: Supplementary Material 11. — Comparison of population attributable fraction (PAF, %) in specific cancer attributed to deficit in physical activity (DPA) when using different relative risks. WHO, World Health Organization; UK, United Kingdom; IPAQ, International Physical Activity Questionnaire. [file epih-47-e2025010-Supplementary-11.pptx]

## Slide 1
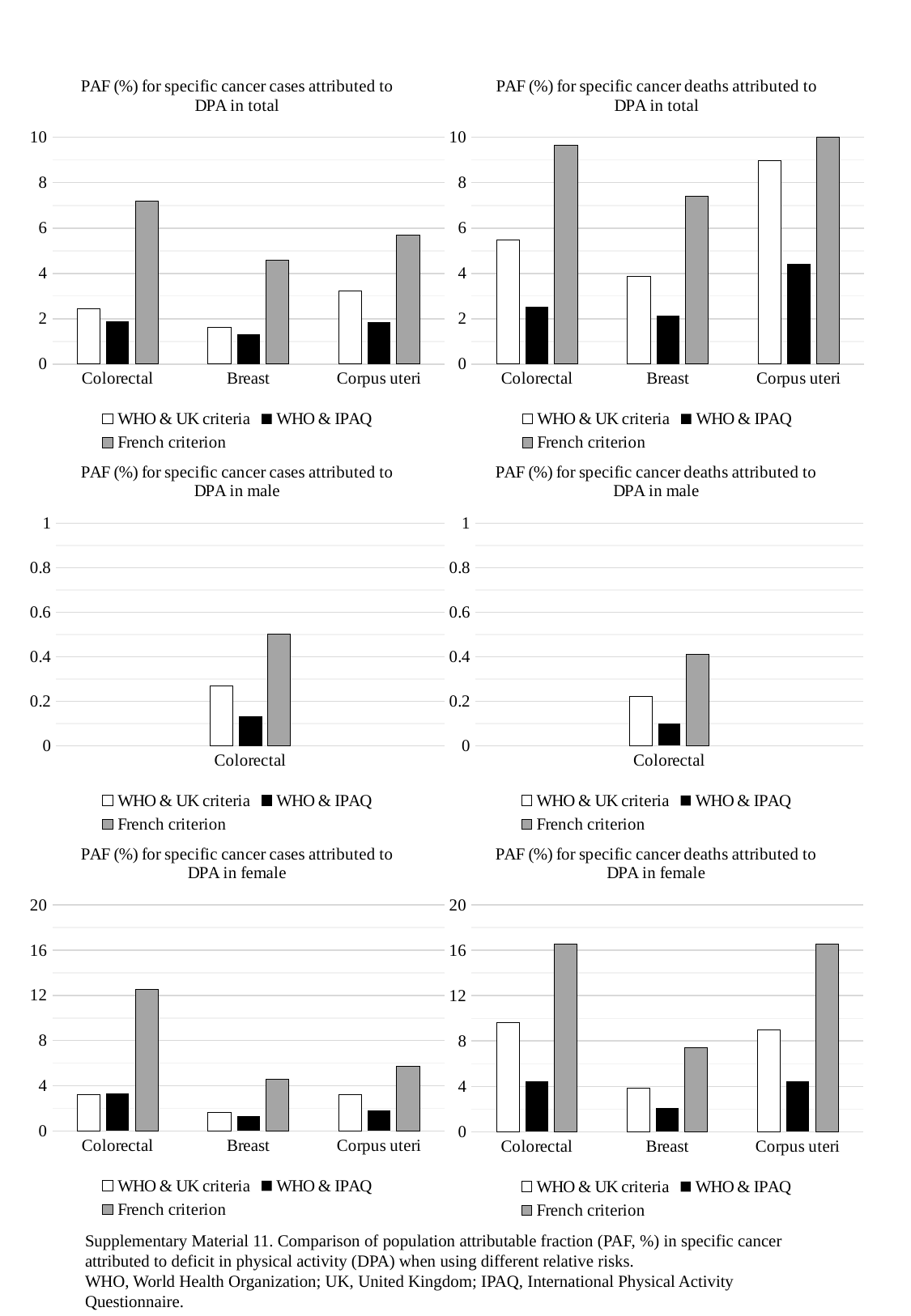

### Chart: PAF (%) for specific cancer cases attributed to DPA in total
| Category | WHO & UK criteria | WHO & IPAQ | French criterion |
|---|---|---|---|
| Colorectal | 2.43 | 1.88 | 7.2 |
| Breast | 1.62 | 1.31 | 4.57 |
| Corpus uteri | 3.22 | 1.84 | 5.68 |
### Chart: PAF (%) for specific cancer deaths attributed to DPA in total
| Category | WHO & UK criteria | WHO & IPAQ | French criterion |
|---|---|---|---|
| Colorectal | 5.47 | 2.54 | 9.64 |
| Breast | 3.86 | 2.12 | 7.39 |
| Corpus uteri | 8.99 | 4.43 | 16.54 |
### Chart: PAF (%) for specific cancer cases attributed to DPA in male
| Category | WHO & UK criteria | WHO & IPAQ | French criterion |
|---|---|---|---|
| | None | None | None |
| Colorectal | 0.27 | 0.13 | 0.5 |
### Chart: PAF (%) for specific cancer deaths attributed to DPA in male
| Category | WHO & UK criteria | WHO & IPAQ | French criterion |
|---|---|---|---|
| | None | None | None |
| Colorectal | 0.22 | 0.1 | 0.41 |
### Chart: PAF (%) for specific cancer cases attributed to DPA in female
| Category | WHO & UK criteria | WHO & IPAQ | French criterion |
|---|---|---|---|
| Colorectal | 3.22 | 3.33 | 12.54 |
| Breast | 1.62 | 1.31 | 4.57 |
| Corpus uteri | 3.22 | 1.84 | 5.68 |
### Chart: PAF (%) for specific cancer deaths attributed to DPA in female
| Category | WHO & UK criteria | WHO & IPAQ | French criterion |
|---|---|---|---|
| Colorectal | 9.6 | 4.43 | 16.54 |
| Breast | 3.86 | 2.12 | 7.39 |
| Corpus uteri | 8.99 | 4.43 | 16.54 |Supplementary Material 11. Comparison of population attributable fraction (PAF, %) in specific cancer attributed to deficit in physical activity (DPA) when using different relative risks.
WHO, World Health Organization; UK, United Kingdom; IPAQ, International Physical Activity Questionnaire.
