# Supplementary material for: Preventable cancer cases and deaths attributable to deficit of physical activity in Korea from 2015 to 2030
Source: Epidemiol Health. 2025 Jan 27;47:e2025010. doi: 10.4178/epih.e2025010 (PMC12531471; doi:10.4178/epih.e2025010)
Supplement: Supplementary Material 12. — Comparison of fraction (%) of deficit in physical activity on cancer in 2015 (15-latency) and in 2009 (19-latency) in Korea [file epih-47-e2025010-Supplementary-12.docx]

Supplementary Material 12. Comparison of fraction (%) of deficit in physical activity on cancer in 2015 (15-latency) and in 2009 (19-latency) in Korea

|  | **Total** | | | | **Men** | | | | **Women** | | | |
| --- | --- | --- | --- | --- | --- | --- | --- | --- | --- | --- | --- | --- |
|  | **2009** | | **2015** | | **2009** | | **2015** | | **2009** | | **2015** | |
|  | **PAF %** | **AC** | **PAF %** | **AC** | **PAF %** | **AC** | **PAF %** | **AC** | **PAF %** | **AC** | **PAF %** | **AC** |
| **Incidence** |  |  |  |  |  |  |  |  |  |  |  |  |
| Colorectal | 0.82 | 202 | 2.43 | 658 | 0.78 | 117 | 1.88 | 303 | 0.87 | 85 | 3.22 | 355 |
| Breast^1^ | 8.81 | 1,176 | 1.62 | 173 |  |  |  |  | 8.81 | 1,176 | 1.62 | 173 |
| Corpus uteri |  |  | 3.22 | 78 |  |  |  |  |  |  | 3.22 | 78 |
| **All cancers** | **0.70** | **1,378** | **0.42** | **909** | **0.10** | **117** | **0.27** | **303** | **1.40** | **1,261** | **0.60** | **606** |
| **Mortality** |  |  |  |  |  |  |  |  |  |  |  |  |
| Colorectal | 0.82 | 58 | 5.47 | 454 | 0.78 | 31 | 2.31 | 109 | 0.87 | 27 | 9.60 | 345 |
| Breast^1^ | 8.81 | 165 | 3.86 | 66 |  |  |  |  | 8.81 | 165 | 3.86 | 66 |
| Corpus uteri |  |  | 8.99 | 29 |  |  |  |  |  |  | 8.99 | 29 |
| **All cancers** | **0.30** | **223** | **0.68** | **548** | **0.10** | **31** | **0.22** | **109** | **0.70** | **192** | **1.42** | **440** |

Abbreviation: PAF, Population attributable fraction; AC, Number of attributable cases; MET, Metabolic equivalent of task.

1. Among postmenopausal female

In 2009, the definition of physical activity was derived from the combination of both frequency and duration of recreational exercises. Physical activity during leisure time was considered low if the routine involved exercising for fewer than 15 minutes per session and less than once a week.

In 2015, engaging in a moderate activity at 6 MET for 30 minutes daily over 5 days, or participating in a vigorous activity at 12 MET for 15 minutes daily over 5 days, results in a total of 900 METs minute per week. According to the criteria of UK, physical activity levels below 900 METs minute per week are defined as insufficient.
